# Thermodynamics of Molecular Transport Through a Nanochannel: Evidence of Energy–Entropy Compensation

**DOI:** 10.3390/ijms26157277

**Published:** 2025-07-28

**Authors:** Changsun Eun

**Affiliations:** Department of Chemistry, Hankuk University of Foreign Studies, Yongin 17035, Republic of Korea; ceun@hufs.ac.kr

**Keywords:** molecular transport, energy–entropy compensation, enthalpy–entropy compensation, EEC, free energy, potential of mean force, molecular dynamics simulation, CNT

## Abstract

In this work, the thermodynamics of molecular transport between two compartments connected by a nanochannel is investigated through an analysis of internal energy and entropy changes, with a focus on how these changes depend on intermolecular interaction strength. When interactions are weak, resembling gas-like behavior, entropy dominates and favors configurations in which molecules are evenly distributed between the two compartments, despite an increase in internal energy. In contrast, strong interactions, characteristic of liquid-like behavior, lead to dominant energetic contributions that favor configurations with molecules localized in a single compartment, despite entropy loss. Intermediate interaction strengths yield comparable entropic and energetic contributions that cancel each other out, resulting in oscillatory behavior between evenly distributed and localized configurations, as observed in previous work. This thermodynamic analysis reveals energy–entropy compensation, in which entropic and energetic contributions offset each other across different interaction strengths; notably, this compensatory relationship exhibits a linear trend. These findings provide insight into the thermodynamic origins of molecular transport behavior and highlight fundamental parallels between molecular transport and molecular binding, the latter being particularly relevant to molecular recognition and drug design.

## 1. Introduction

Molecular transport through channels is a fundamental mechanism that underpins various biological and technological processes, including ion conduction through membrane ion channels [1,2,3], the translocation of ribonucleic acids (RNAs) and proteins through nuclear pore complexes [4,5,6,7,8], and selective molecular separation in nanofluidic systems [9,10,11,12]. Regulating such transport—i.e., controlling the rate and direction of molecular flow through a channel—requires a clear understanding of both thermodynamic and kinetic factors. To investigate these aspects, we previously studied molecular transport in a minimal model system consisting of two compartments connected by a carbon nanotube (CNT), where small molecules moved between the compartments according to the thermodynamics of the system [13,14,15].

Extensive molecular dynamics (MD) studies have shown that simplified nanochannel models—particularly those based on CNTs and graphenes—can effectively capture key microscopic aspects of molecular behavior under confinement. These studies have revealed a wide range of behaviors, including collective motion of confined water [16,17,18], ultrafast flow rates of water [19,20,21], and sensitivity to confinement geometry and interactions. Specific factors investigated include water–wall interactions [22,23], pore size [24,25], CNT length [26], CNT defects [27], entrance-region water structure [28], partial charges on CNT atoms [29], osmotic effects [30,31,32,33], electric field effects [34,35,36,37,38], the potential of mean force (PMF) for water translocation in pure water [29,39] and ionic solutions [11,40], functionalization of a CNT [39], and the thermodynamics of single-file water loading [41]. A few review articles further underscore the scope and depth of insight gained from MD simulations in nanochannels [42,43,44,45]. Building on the conceptual value of these studies, we focus here on thermodynamic decomposition and energy–entropy compensation as a complementary route to understanding molecular transport.

As a foundation for the present analysis, we briefly summarize key findings from our previous studies [13,14,15], in which we used our compartment-based nanochannel model to calculate the free energy change associated with transferring all molecules from one compartment to the other. We found that the shape of the free energy profile strongly depends on the strength of intermolecular interactions.

When interactions are weak, the molecules behave in a gas-like manner and tend to distribute evenly between the two compartments. In this case, the free energy decreases as the system evolves from a state in which all molecules reside in one compartment to a state with an even distribution, and then increases as the molecules move to the opposite compartment—resulting in a free energy well centered at the midpoint of the free energy profile [14,15].

In contrast, strong interactions lead the molecules to aggregate and resist separation, thereby impeding transport. The free energy increases as molecules begin to redistribute, reaches a maximum at the midpoint (where the molecules are evenly distributed across the two compartments), and then decreases—resulting in a barrier centered in the profile [13,15].

At intermediate interaction strengths, the free energy profile exhibits a relatively flat region around the center, indicating negligible free energy changes as the molecules redistribute. This suggests the possibility of a reversible transport process involving many molecules, which was confirmed by our unconstrained MD simulations [15].

In the present study, we build upon this prior work by decomposing the free energy into its energetic and entropic contributions. We also explore potential correlations between these two contributions—a topic of longstanding interest in thermodynamic studies.

Experimentally, the relationship between enthalpic (or energetic) and entropic changes has been extensively studied in a wide range of cases, including chemical reactions [46,47,48], molecular recognition processes (e.g., ligand–protein binding and molecular docking) [49,50,51,52,53,54,55,56,57], protein unfolding [52,58,59], and solvation [52,60]. In many cases, a linear correlation—commonly referred to as the enthalpy–entropy compensation (EEC) effect—has been observed, in which opposing contributions due to the changes in enthalpy and entropy offset one another [54,55,61,62]. A related concept in kinetics is the isokinetic relation [61,63]. In some instances, the compensation is nearly perfect, meaning that the enthalpic and entropic contributions are comparable in magnitude, leading to a net free energy change close to zero. This effect can hinder attempts to improve binding affinity through enthalpic optimization in drug design scenarios [54,64].

Although deviations from compensation behavior have also been reported—including weak or no correlation or even parallel changes in the enthalpic and entropic contributions (“anticompensation”) [62,65,66,67,68,69]—the basic thermodynamic rationale behind EEC remains compelling. Specifically, molecular association typically lowers enthalpy due to favorable interactions but also reduces entropy due to restricted translational, rotational, or conformational freedom in the associated state. These opposing contributions to the total free energy change can cancel each other. However, when solvent effects are involved—especially with water—the mechanisms underlying compensation become more complex [57,61,68,70,71]. Additionally, some studies have questioned the validity of the effect, suggesting it may arise from experimental uncertainties and the mathematical coupling of thermodynamic parameters rather than reflecting a genuine physical phenomenon [52,72,73,74].

Despite continued investigations, the generality and physical origin of the EEC effect remain under debate. In this study, we address this issue in the context of molecular transport through a nanochannel and draw an analogy with molecular binding. Specifically, we define the associated state as one in which all molecules are aggregated in a single compartment, and the dissociated state as one in which molecules are evenly split between the two compartments, with equal amounts on either side of the CNT. This analogy enables us to explore thermodynamic compensation in transport using concepts from molecular binding. Additionally, we identify two features of our system that have not been widely explored in previous molecular binding studies.

The first feature is that our model explicitly includes solvent molecules, whose interactions range from nonpolar to water-like. Given the critical role of solvent effects in biological and chemical systems, it is important to examine the energetic and entropic changes that occur when solvent molecules themselves undergo large-scale aggregation and dissociation. Unlike studies that treat the surrounding solvent environment implicitly or neglect it, our model captures solvent-driven thermodynamic contributions directly. Such background effects are increasingly recognized as essential for understanding compensation phenomena in solution-phase processes [75,76].

The second feature concerns the investigation of the compensation effect occurring in a many-body system. Prior studies have mainly focused on two-body interactions—either explicitly or effectively pairwise—between atoms, molecules, or complexes. In contrast, our model involves collective association and dissociation of many molecules with explicit many-body interactions. This raises the question of whether the compensation effect persists in such aggregation processes, particularly as the interaction strength varies. Since the interaction strength influences phase behavior and can even induce phase transitions [77], examining its role provides valuable thermodynamic insights. Understanding compensation in this many-body context may offer new perspectives on how solvent and many-body effects modulate binding affinity.

## 2. Materials and Methods

### 2.1. Model System

**Figure 1:**
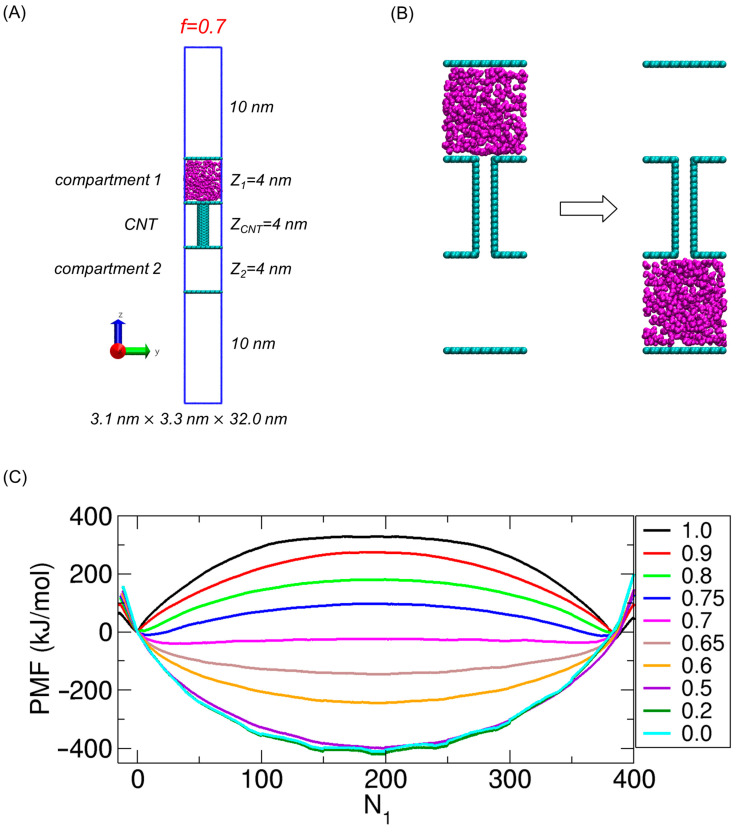
(**A**) Transport model system for water-shaped molecules with *f* = 0.7. Note that the blue lines indicate the periodic boundary of the system. (**B**) Initial and final states of molecular transport process between two compartments through a CNT (carbon nanotube). (**C**) PMF (potential of mean force) profiles as a function of the number of molecules in Compartment 1 (N_1_) for various values of f. Panel (**C**) is adapted from Ref. [15]. © 2025 The Author(s). Published by the Royal Society of Chemistry. Licensed under CC BY 3.0.

In our previous works [13,14,15], we performed MD simulations of molecular transport under constant number, volume, and temperature (NVT) conditions at T = 300 K using a model system consisting of two compartments connected by a carbon nanotube (CNT). In the present study, no new simulations are conducted; instead, we reanalyze the previously generated simulation data to perform thermodynamic analysis for the same system, which is described in the remainder of this section. The CNT had a diameter barely sufficient to accommodate a single molecule, thereby enabling single-file transport. In that system, 400 small molecules were allowed to move between the compartments through the CNT, as illustrated in Figure 1A. The molecular configurations shown in Figure 1A and throughout this paper are prepared using VMD (Visual Molecular Dynamics, version 1.9.4) [78]. To model the compartments and the CNT, we used graphene-like plates with xy dimensions of 3.1 nm × 3.3 nm and a (6,6) armchair CNT with a length of 4 nm. The compartments and the CNT were fixed in space, while only the transported molecules could move.

To study the effect of interaction strength on transport behavior, we employed small molecules with the same molecular geometry as that of the TIP3P water model [79] but with various electrostatic interaction strengths. The interaction strength was modulated by applying a charge scaling factor f, which multiplied the partial charges of the atoms in the TIP3P water molecule [15]. For example, when f= 0.7, the charges on the oxygen and hydrogen atoms became −0.5838 and 0.2919, respectively; these values were obtained by multiplying the original TIP3P charges (−0.834 for O and 0.417 for H) by 0.7. In this way, f= 1 corresponded to water molecules, whereas f= 0 represented nonpolar water-shaped molecules. Note that only these mobile molecules carried partial charges; thus, electrostatic interactions occurred exclusively among them. Long-range electrostatic interactions were treated using the particle–mesh Ewald (PME) method [80]. The cutoff distance for electrostatic interactions was set to 1.4 nm.

For the Lennard–Jones (LJ) interactions, the LJ parameters for the carbon atoms in the CNT were taken from the AMBER force field [81]. The same AMBER carbon type was used for the atoms in the graphene-like plates, but with a reduced ε parameter (the potential well depth) to prevent the adsorption of molecules onto the compartment walls [15,32]. LJ interactions between dissimilar atom types were calculated using the Lorentz–Berthelot mixing rule [82,83]. Periodic boundary conditions were imposed in all three Cartesian directions. The cutoff distance for the LJ interactions was 1.4 nm.

### 2.2. PMF Calculations and Results from Our Previous Work

In our previous work [15], we calculated the free energy change associated with the transport of molecules from one compartment to the other at various values of f (see Figure 1B). Specifically, the free energy change—i.e., the potential of mean force (PMF)—was calculated as a function of the number of molecules in Compartment 1 ($N_{1}$) by applying the weighted histogram analysis method (WHAM) [84,85] to umbrella sampling data obtained from MD simulations under NVT conditions. The simulations were performed at 300 K using the GROMACS package (version 2023.3) [86]. To maintain the temperature, we used a V-rescale thermostat [87] with a coupling constant of 0.1 ps. The integration time step was 2.0 fs. Periodic boundary conditions (PBCs) were applied along all three Cartesian directions.

The basic idea of our PMF method was that molecules must pass through the CNT to be transported from one compartment to the other. When a molecule moved within the CNT, some of the other molecules in the single-file chain could be displaced into one of the compartments, thereby altering $N_{1}$. Therefore, by calculating the PMF for the molecule’s translation through the CNT—a standard PMF setup using umbrella sampling—we indirectly determined the PMF as a function of $N_{1}$.

To account for the complete transport of all molecules, we repeated this PMF calculation for many such molecular translations. The maximum number of molecules displaced by a single translation could reach 10 to 15, depending on *f*, due to the finite capacity of the CNT. For each value of f, we selected 72 different molecules for translation, and accordingly performed 72 independent PMF calculations, resulting in a total of 6144 umbrella sampling windows. For each window, we conducted a 1.5 ns MD simulation with a harmonic umbrella potential using a force constant of 3000 kJ/mol/nm^2^. From each trajectory, we used the final 1 ns and sampled molecular configurations every 5 ps, yielding 201 configurations per window for subsequent analyses. Additionally, for the WHAM calculation, the coordinates and forces of the molecule selected for the umbrella potential were saved more frequently—every 0.1 ps—to ensure sufficient sampling resolution along the reaction coordinate.

After obtaining the 72 partial PMF segments covering different $N_{1}$ intervals, we merged them to construct a single PMF spanning the full range of $N_{1}$. Note that $N_{1}=$ 0 corresponds to the state in which no molecules are present in Compartment 1, but the CNT is fully occupied. To account for cases where the CNT is not completely filled—which was necessary for complete transport—we introduced negative $N_{1}$ values; for example, $N_{1}$ = −1 indicates that one molecule is missing from full-CNT occupancy. The minimum $N_{1}$ value ranged from −10 to −15, depending on *f*. The state at $N_{1}=$ 0 was chosen as the reference, with its PMF value set to zero. The resulting PMF profiles are shown in Figure 1C. Further simulation and methodological details are provided in our previous work [15].

**Figure 2:**
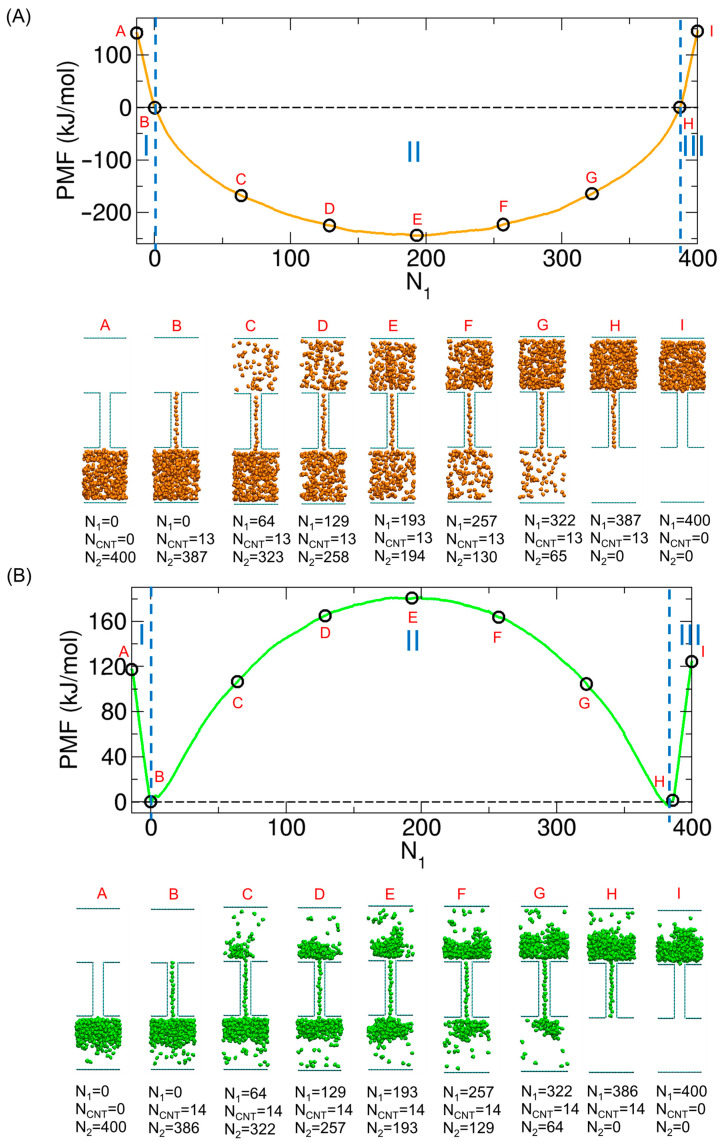
(**A**) Characteristic states denoted by A through I along the PMF (potential of mean force) profile for f= 0.6 (**top**, orange curve from Figure 1C) and the corresponding molecular configurations (**bottom**). (**B**) Characteristic states denoted by A through I along the PMF profile for f= 0.8 (green curve) and the corresponding configurations. Reproduced from Ref. [15]. © 2025 The Author(s). Published by the Royal Society of Chemistry. Licensed under CC BY 3.0.

To better understand the PMF profiles in Figure 1C, we previously analyzed the molecular configurations corresponding to key states along the PMF curves for two representative cases—weak electrostatic interactions (f= 0.6) and strong interactions (f= 0.8)—as shown in Figure 2A,B, respectively. For f= 0.6, the PMF had a minimum at the state where the molecules were evenly distributed between the two compartments (State E). Thus, starting from a state where all molecules resided in Compartment 1 (State I), the free energy decreased as molecules were transported to Compartment 2, reaching a minimum at State E. Further transport to Compartment 2 led to an increase in the free energy.

Similarly, for f= 0.8, transport spontaneously proceeded from the initial state (State I) to State H, where the molecules filled the inner space of the CNT. However, further transport from State H to State E did not occur spontaneously, as it resulted in an increase in the PMF. Once the system reached State E—after transporting approximately half of the molecules—transport continued spontaneously until State B was reached. Finally, since the molecules confined within the CNT were thermodynamically stable, extracting them from the CNT to complete the transport increased the free energy.

Based on this analysis, we identified three distinct regimes in the PMF profiles, labeled I, II, and III in Figure 2, according to the characteristic behavior of the system. When considering the transport of molecules from Compartment 1 to Compartment 2 (see Figure 1B), Regime III corresponded to the initial filling of molecules into the CNT, which was thermodynamically favorable. Regime I represented the final stage in which molecules exited the CNT, a process that was thermodynamically unfavorable. The thermodynamics associated with Regimes III (loading, entering) and I (unloading, exiting) have been examined in detail in a previous study using free energy calculations based on thermodynamic integration [41], although the model employed there differs from ours. Regime II described the states in which the CNT was fully occupied, and molecules were transported between the compartments. The behavior in Regime II strongly depended on the interaction strength. For f= 0.6, the PMF exhibited a well in this regime, with a minimum near the center (State E) and maxima at the ends (States B and H), as shown in Figure 2A. For f= 0.8, the opposite situation occurred: the PMF exhibited a barrier with a maximum at the evenly distributed state (State E) and minima at the boundaries of Regime II (States B and H), as shown in Figure 2B.

**Figure 3:**
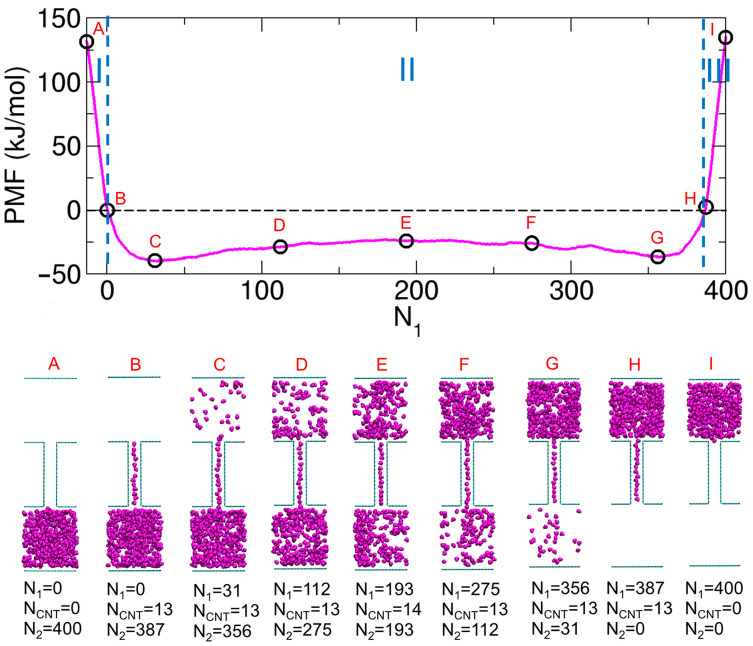
Characteristic states denoted by A through I along the PMF (potential of mean force) profile for f= 0.7 (**top**, magenta curve from Figure 1C) and the corresponding molecular configurations (**bottom**).

The distinct change in the shape of the PMF with varying *f* raised the question of how the profile transitioned from the weak-interaction case (f= 0.6) to the strong-interaction case (f= 0.8). To address this question, we referred to the PMF profile for f= 0.7. Although our previous work [15] did not include a figure showing the characteristic configurations for f= 0.7 (as in Figure 2), we present them here for consistency and further discussion. As shown in Figure 3, when f= 0.7, the PMF exhibits an almost flat region where there was a well for f= 0.6 and a barrier for f= 0.8. We also identify representative configurations along the PMF profile for f= 0.7. Together with the cases of f= 0.6 and 0.8, the profile for f= 0.7 reveals a consistent trend in Regimes I and III, whereas the distinguishing feature appears in Regime II.

In Regime II, at intermediate interaction strengths (e.g., f= 0.7), the cancelation between the central barrier (from the strong-interaction case) and the central well (from the weak case) gave rise to a shallow barrier located at State E, flanked by two shallow wells located at States C and G. This resulted in an almost flat region in the free energy profile near the center. Due to this flatness, transitions between the states occurred with negligible free energy costs, enabling the transport of a large number of molecules. Indeed, we observed frequent transitions between states near C and states near G in unconstrained MD simulations, resulting in large fluctuations in $N_{1}$ [15].

Based on the discussion above, we proposed that f= 0.7 serves as a threshold that determines the shape of the PMF profile in Regime II. Specifically, when f> 0.7, the profile exhibited a barrier in Regime II, and we referred to this range (f> 0.7) as the water-like or strong-interaction regime [15]. Conversely, when f< 0.7, the profile contained a well in Regime II, which we referred to as the nonpolar-like or weak-interaction regime [15].

For later discussion, we now define two representative molecular distributions in Regime II: the single-compartment–full-CNT state and the evenly distributed state. In the single-compartment–full-CNT state (States B or H), the CNT is fully occupied, and the remaining molecules reside in only one compartment. In contrast, the evenly distributed state (State E) corresponds to a configuration in which the molecules are equally divided between the two compartments, with the CNT also fully occupied. To improve clarity, we also introduce a notation $S_{N_{2}}^{N_{1}}$, where $N_{1}$ and $N_{2}$ denote the number of molecules in Compartments 1 and 2, respectively. The superscript and subscript are chosen to match the vertical arrangement in Figure 1A, where Compartment 1 is at the top and Compartment 2 is at the bottom. Since the total number of molecules is fixed at 400, the number of molecules in the CNT ($N_{CNT})$ is implicitly defined as 400$-N_{1}-N_{2}$. Using this notation, the states labeled A, B, E, H and I in Figure 3 correspond to $S_{400}^{0}$, $S_{387}^{0}$, $S_{193}^{193}$, $S_{0}^{387}$, and $S_{0}^{400}$, respectively.

### 2.3. Thermodynamic Analysis

In general, the PMF represents the change in free energy along a chosen coordinate. In this study, the relevant free energy is the Helmholtz free energy (A), which is the natural thermodynamic potential under NVT conditions, and the coordinate is $N_{1}$, as illustrated in Figure 1C. To quantify how much energetic and entropic changes contribute to the total free energy, we use the thermodynamic relation ∆A=∆U−T∆S, where U and S denote internal energy and entropy, respectively.

Moreover, the internal energy change can be further decomposed into the changes in kinetic and potential energies: ∆U=∆KE+∆PE. In this relation, the change in kinetic energy (∆KE) is negligible because the simulation temperature is fixed at 300 K. Therefore, we approximate ∆U≅∆PE, meaning that the internal energy change effectively corresponds to the change in potential energy. Accordingly, the entropic contribution is obtained as the difference between the free energy change and the potential energy change, i.e., −T∆S≅∆A−∆PE. In this way, we decompose the PMF into energetic (∆U) and entropic (−T∆S) contributions.

This decomposition is carried out numerically using simulation data, rather than being derived analytically from partition functions. It captures the net thermodynamic contributions to free energy but does not separately resolve contributions from specific entropy sources such as vibrational, rotational, or configurational entropy.

To compute the energetic and entropic contributions, we calculate the potential energy of the model system as a function of $N_{1}$ for representative values of f, in order to examine how the internal energy changes as molecules move from one compartment to the other. For this analysis, we use the MD simulation trajectories corresponding to various $N_{1}$ states that were previously generated for the PMF calculations reported in our earlier work [15]. Therefore, as noted in Section 2.1, we do not perform any new MD simulations in this study; rather, we analyze data from those PMF calculations. As mentioned in Section 2.2, in those PMF calculations, we employed a total number of 6144 umbrella sampling windows for each value of f. For each window, a 1.5 ns MD simulation was performed, and the last 1 ns of the trajectory was used for both the previous PMF and the present potential energy analysis. For the potential energy analysis, molecular configurations were sampled every 5 ps, $N_{1}$ esulting in 201 configurations per window.

Since the relative magnitude of potential energy is physically meaningful, we compute the relative potential energy by shifting the original values along the *y*-axis such that the running average calculated over 10 points at $N_{1}$ 0—where no molecules are present in Compartment 1 and the CNT is fully occupied (State B in Figure 2 and Figure 3)—is set to zero, consistent with the reference value used in the PMF profiles. At this reference point, ∆A= 0, ∆U= 0, and therefore −T∆S= 0. These calculations are performed using the gmx tool of the GROMACS package (version 2023.3) [86], and the resulting plots are generated using the xmgrace program (version 5.1.25; https://plasma-gate.weizmann.ac.il/Grace/ (accessed on 27 October 2017)), as are the other figures presented in this study.

After obtaining the energetic contribution from the potential energy change (∆U≅∆PE), we calculate the entropic contribution (−T∆S) by subtracting the energetic term from the free energy change (∆A or PMF) obtained in our previous work [15]. Based on this composition, we then analyze the correlation between the energetic and entropic contributions in the present study.

## 3. Results and Discussion

### 3.1. Total Potential Energy Profiles

**Figure 4:**
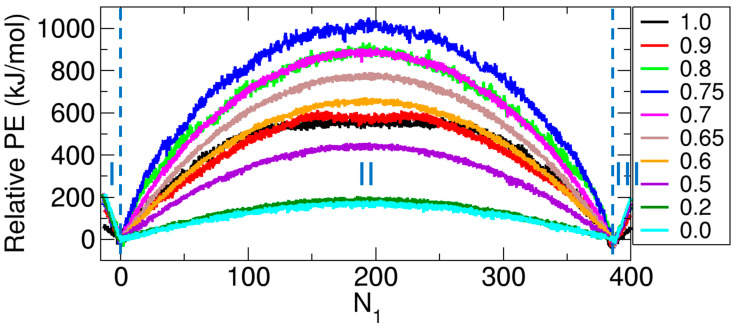
Relative potential energy (Relative PE) as a function of $N_{1}$ for various values of f. All Relative PE values are defined with respect to those at $N_{1}=\;$0; that is, all energies at $N_{1}=\;0$ are set to zero. The profiles are constructed by shifting the data such that the running average calculated over 10 points at $N_{1}=$ 0 is set to zero.

The results of the potential energy calculations are shown in Figure 4. In contrast to the PMF profiles shown in Figure 1C, the potential energy profiles do not exhibit wells regardless of the value of f; instead, they consistently display a barrier. Specifically, the relative potential energy reaches its minima at the single-compartment–full-CNT states (States B and H, corresponding to $S_{385}^{0}-S_{390}^{0}$ and $S_{0}^{385}-S_{0}^{390}$, respectively, depending on f) and a maximum at the evenly distributed state (State E, expressed as $S_{195}^{195}-S_{192}^{193}$, with $S_{192}^{193}=S_{193}^{192}$ by symmetry) in most cases. Only at f= 0.9 and 1.0 does the maximum deviate slightly from State E, which may be attributed to the energetic stabilization of that state.

Importantly, the interaction strength, modulated by f, significantly affects the height of the potential energy barrier. The barrier height increases with f up to f= 0.75 but decreases beyond this point, showing a nonmonotonic trend. This behavior appears to be associated with a phase-like transition in molecular organization near f≈ 0.7. At low values of f (i.e., f< 0.7), the molecules behave in a gas-like manner. Thus, when they are more dispersed—as in the evenly distributed state (State E, or $S_{194}^{193}$ in Figure 2A)—the potential energy increases due to the lack of stabilizing interactions. Consequently, the barrier height at the evenly distributed state increases with f in this regime.

However, at higher values of f (i.e., f> 0.7), the molecules in the *evenly distributed* state (State E, or $S_{193}^{193}$ in Figure 2B) tend to aggregate within each compartment, leading to enhanced stabilization reminiscent of water-like behavior. This aggregation effect reduces the potential energy difference between the evenly distributed state (State E) and the single-compartment–full-CNT state (State B or H, or $S_{386}^{0}$ or $S_{0}^{386}$ in Figure 2B), thereby lowering the barrier height. This effect becomes more pronounced as f increases.

Notably, the maximum barrier height occurs at f= 0.75 rather than at f= 0.7, and the barrier becomes flatter at f= 0.9 and 1.0. These observations reflect the complexity of molecular interactions under strong-interaction conditions. Additionally, at f≈ 0.7, the largest fluctuations in $N_{1}$ were observed in our previous work [15], with the molecules appearing disordered or irregularly distributed within the compartments (see Figure 3), making it difficult for them to interact effectively in such an irregularly distributed configuration. This behavior in the phase transition regime may further contribute to the observed deviation in the potential energy profiles, where the maximum barrier height occurs at f= 0.75 rather than at the expected transition point of f= 0.7.

### 3.2. Decomposition of Total Potential Energy by Interaction Type

**Figure 5:**
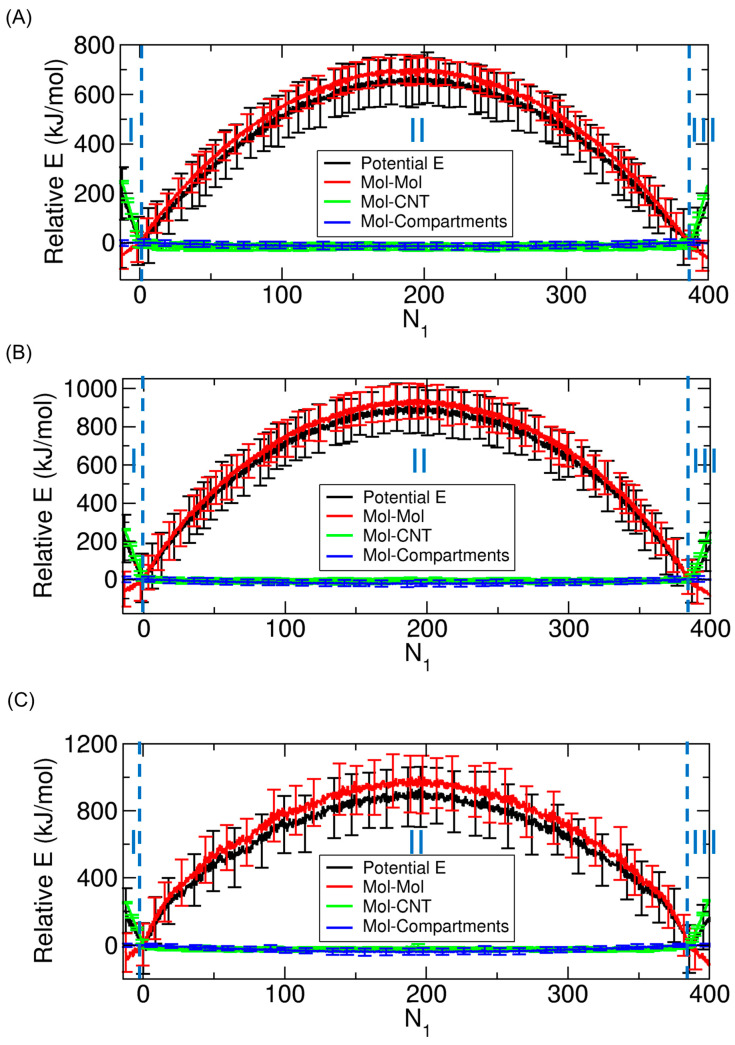
Decomposition of the potential energy profiles (Potential E; black) shown in Figure 4 as a function of $N_{1}$ for (**A**) f=0.6, (**B**) f= 0.7, and (**C**) f= 0.8. The energies presented here are relative energies (Relative E) defined with respect to those at $N_{1}=\;$0; that is, all energies at $N_{1}=\;0$ are set to zero. Each profile is decomposed into three components: interactions among the transported molecules (Mol–Mol; red), interactions between the molecules and the CNT (carbon nanotube) (Mol–CNT; green), and interactions between the molecules and the compartments (Mol–Compartments; blue). Error bars represent standard deviations.

A key question is which type of interaction dominates the observed changes in potential energy. To address this, we decompose the total potential energy into three components, each corresponding to a distinct type of interaction: between the transported molecules and the CNT, between the molecules and the compartments, and among the molecules themselves. The results for three representative cases (f= 0.6, 0.7, and 0.8) are shown in Figure 5.

Regardless of the value of f, the decomposition clearly reveals distinct dominant interactions across the three regimes. In Regimes I and III, where molecules enter or exit the CNT, the dominant contribution to the change in potential energy arises from CNT–molecule interactions. In contrast, in Regime II, where molecules move between the compartments, the potential energy change is primarily due to intermolecular interactions among the transported molecules. In all regimes, the contribution from molecule–compartment interactions remains minimal and nearly constant throughout. These results indicate that the energetic changes associated with molecular filling and exiting processes are governed by CNT–molecule interactions, whereas the transport process between compartments is mainly influenced by intermolecular interactions.

### 3.3. Decomposition of the PMF into Energetic and Entropic Contributions

Having obtained the PMF profiles (see Figure 1C) and potential energy profiles (see Figure 4), a natural question arises: in weak-interaction cases (f< 0.7), how can the PMF profile exhibit a well centered at the evenly distributed state (State E, or $S_{194}^{193}$ in Figure 2A) while the corresponding potential energy profile shows a barrier? This observation suggests that the driving force toward State E is not potential energy (or internal energy), but rather entropy. What about other interaction strengths—are they primarily driven by potential energy or by entropy?

**Figure 6:**
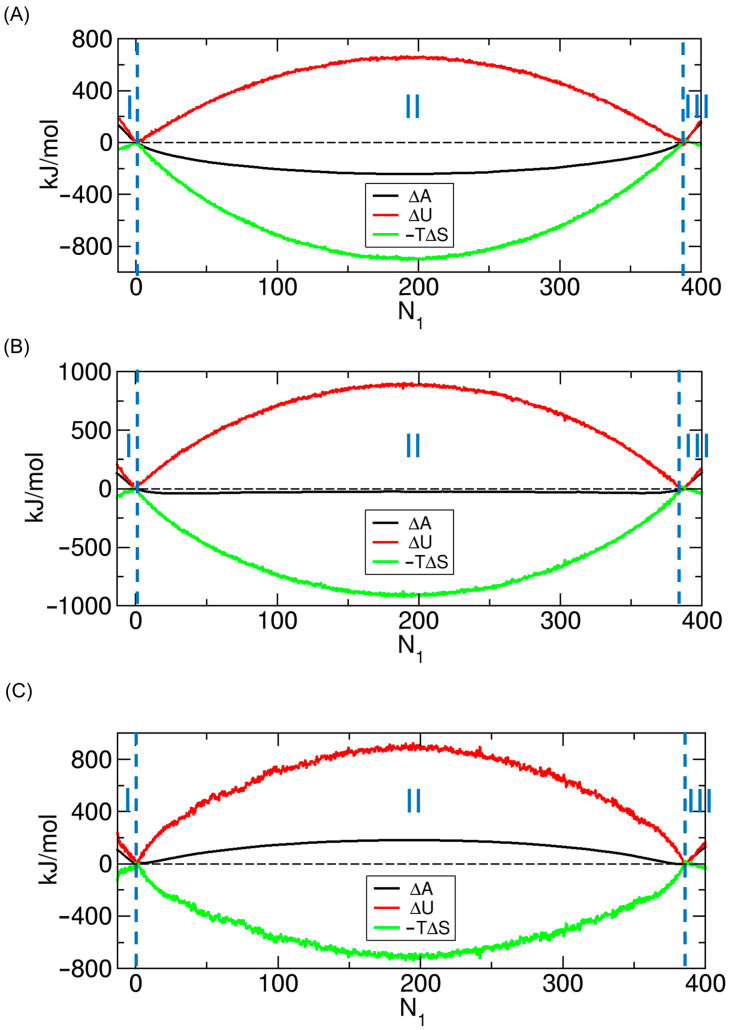
Decomposition of the PMF (potential of mean force, ∆A; black) from Figure 1C into its energetic (∆U; red) and entropic (−T∆S; green) contributions for (**A**) f= 0.6, (**B**) f= 0.7, and (**C**) f= 0.8. The energetic contribution (∆U) corresponds to the potential energy profile (∆PE) shown in Figure 4, as ∆U is very closely approximated by ∆PE under constant temperature. Note that ∆A=∆U−T∆S, and thus the sum of ∆U and −T∆S equals ∆A.

To address this question more broadly, we compute the energetic and entropic contributions to the PMF, as described in Section 2.3. The results of this decomposition are shown in Figure 6. These results reveal that the energetic (potential energy) and entropic contributions to the PMF oppose each other. Specifically, in weak-interaction cases (f< 0.7), the entropic contribution dominates, whereas in strong-interaction cases (f> 0.7), the energetic contribution prevails. Interestingly, at an intermediate (f= 0.7), the two contributions are nearly equal and effectively cancel each other, resulting in a PMF profile with an almost flat region (see Figure 3). Thus, returning to the earlier question, the PMF exhibits a minimum at the evenly distributed state (State E) under weak-interaction conditions (f< 0.7) because the thermodynamically favorable entropic contribution more than compensates for the unfavorable energetic contribution (see Figure 6A).

Another important feature of Figure 6 is that the energetic (red) and entropic (green) contributions consistently exhibit opposite signs, such that an increase in one is accompanied by a decrease in the other. This opposing behavior along changes in $N_{1}$—which may be viewed as a perturbation to the system—is indicative of the energy–entropy compensation effect, which is further discussed in Section 3.4, Section 3.5 and Section 3.6.

### 3.4. Free Energy, Energetic, and Entropic Costs of Molecular Transport

In this study, we focus on the main stage of molecular transport between the two compartments, corresponding to Regime II in the PMF profiles. Specifically, we examine the transport process in which the CNT is fully occupied by molecules, and the transition occurs between two single-compartment–full-CNT states via the evenly distributed state. For example, transport from Compartment 1 to Compartment 2 follows the sequence State H → State E → State B in Figure 2.

Under weak-interaction conditions (f< 0.7), the transition from State H (or $S_{0}^{387}$ in Figure 2A) to State E (or $S_{194}^{193}$) proceeds spontaneously, as the PMF decreases. In contrast, the subsequent transition from State E to State B (or $S_{387}^{0}$) is not spontaneous. In this case, the system must escape from a free energy well centered at State E—i.e., it must climb out of the well to reach State B. Accordingly, the free energy cost is defined as the depth of this well, given by the free energy difference between State B and State E.

In contrast, under strong-interaction conditions (f> 0.7), the direction of spontaneity is reversed. The transition from State H (or $S_{0}^{386}$ in Figure 2B) to State E (or $S_{193}^{193}$) is not spontaneous, while the transition from State E to State B (or $S_{386}^{0}$) is. Thus, the free energy cost corresponds to the height of the barrier between State E and State H.

For the intermediate interaction case (f= 0.7), the PMF profile appears nearly flat overall but exhibits both barrier-like and well-like features: two shallow wells at States C (or $S_{356}^{31}$ in Figure 3) and G (or $S_{31}^{356}$), and a shallow barrier at State E (or $S_{193}^{193}$). In this scenario, although one could define the free energy cost based on the transition between States B (or $S_{387}^{0}$) and H (or $S_{0}^{387}$) as in the weak and strong cases, we instead focus on the reversible transport process between States C and G. This process is more likely to occur in practice, as observed in unconstrained MD simulations in our previous work [15]. Therefore, in the intermediate case, we define the free energy cost as the height of the barrier between State C and State G.

In all cases, completing the transport of all molecules from one compartment to the other requires overcoming a free energy barrier or escaping from a free energy well, both of which entail a thermodynamic cost. Accordingly, we define the free energy cost as the barrier height (for strong and intermediate interactions) or the well depth (for weak interactions).

Due to the left–right symmetry of the PMF profiles and the lack of interest in the directionality of the transport, we compute both $∆A\left(E\right)-∆A\left(H\right)$ and $∆A\left(E\right)-∆A\left(B\right)$ and take their average to define the signed free energy cost (∆∆A, or simply $∆A_{cost}$), where $∆A_{cost}$ is negative for a well and positive for a barrier. In general, the term “free energy cost” refers to the absolute value of $∆A_{cost}$. At intermediate strength (f= 0.7), we replace $∆A\left(H\right)$ and $∆A\left(B\right)$ with $∆A\left(G\right)$ and $∆A\left(C\right)$, respectively, in calculating $∆A_{cost}$.

**Figure 7:**
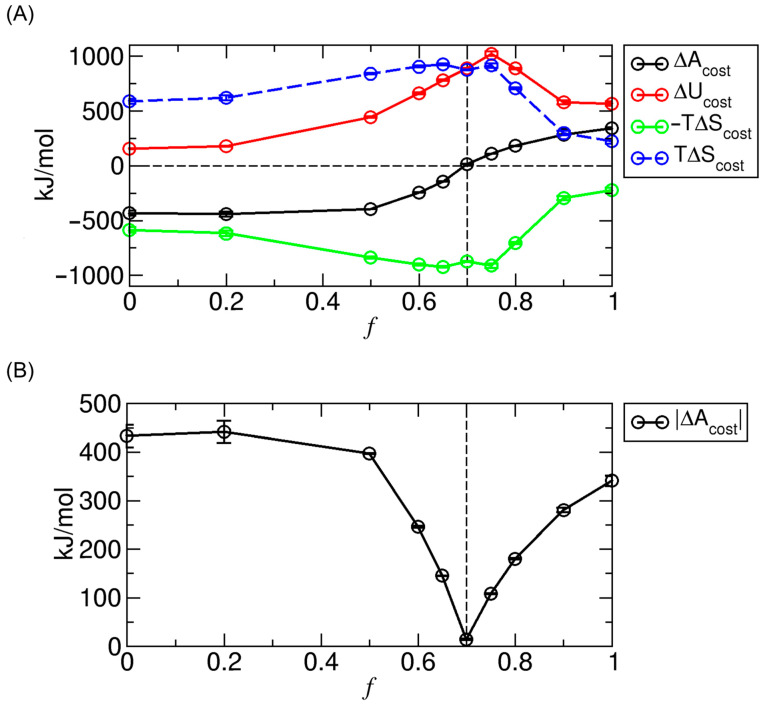
(**A**) Signed free energy cost ($∆A_{cost}$; black) for molecular transport in Regime II of the PMF (potential of mean force) profiles, along with its decomposition into energetic ($∆U_{cost}$; red) and entropic ($-T∆S_{cost}$; green) components. For comparison of magnitudes, the absolute value of the entropic cost ($\left|-T∆S_{cost}\right|$ or $\;T∆S_{cost}$; blue) is also shown. Error bars represent standard deviations. (**B**) Magnitude of the free energy cost required to either escape from the PMF well (f< 0.7) or overcome the PMF barrier (f≥ 0.7) during molecular transport from one compartment to the other in Regime II.

In addition to the signed free energy cost ($∆A_{cost}$), we also decompose it into its energetic ($∆U_{cost}$) and entropic ($-T∆S_{cost}$) components, based on the contributions calculated in Section 3.3. These values represent the corresponding barrier heights or well depths in the energetic and entropic profiles. The results of this decomposition are shown in Figure 7A.

As shown in Figure 7A, in the weak-interaction regime (f< 0.7), the negative entropic contribution ($-T∆S_{cost}<\;$0) dominates over the positive energetic component ($∆U_{cost}>\;$0), resulting in $∆A_{cost}<\;$0 (i.e., a well). In the strong-interaction regime (f> 0.7), the positive energetic contribution is dominant, producing a free energy barrier. In the intermediate case (f= 0.7), the negative entropic and positive energetic contributions nearly cancel each other, yielding $∆A_{cost}\approx 0$.

To better visualize how the magnitude of the free energy cost varies with interaction strength, we plot $\left|∆A_{cost}\right|\;$ as a function of f in Figure 7B. The results show that the cost is generally higher in the gas-like phase (f< 0.7) than in the liquid-like phase (f> 0.7) and remains minimal at the transition point (f= 0.7).

### 3.5. Analysis of Energy–Entropy Compensation Across Interaction Strengths

The molecular transport process in our model system involves changes in the thermodynamic state—specifically, in internal energy, entropy, and ultimately free energy—as shown in Figure 6 and Figure 7. Interestingly, in both figures, the energetic and entropic contributions consistently exhibit opposite signs; for example, $∆U_{cost}>\;$0 and $-T∆S_{cost}<\;$0 in Figure 7, suggesting a compensation between them. In this section, we systematically investigate whether a correlation, including compensation, exists between these two contributions across different values of the interaction strength f, and, if so, how the degree of compensation depends on f.

Before examining the correlation in detail, we first consider the physical meaning of the compensation observed in Figure 7, where again, $-T∆S_{cost}$ (green) is consistently negative and $∆U_{cost}$ (red) is consistently positive. The fact that $-T∆S_{cost}<\;$0 implies that $∆S_{cost}>$ 0, indicating an increase in entropy during the transition from the single-compartment–full-CNT states (States B or H, or for f= 0.7, States C or G) to the evenly distributed state (State E). This is physically reasonable: the dispersed molecular configuration in State E has higher entropy than the more compact configurations in the other states (see Figure 2 and Figure 3). In contrast, $∆U_{cost}>$ 0 indicates an increase in internal energy during the same transition, as molecules in more compact configurations experience stronger intermolecular interactions than in the dispersed configuration of State E.

Taken together, the redistribution of molecules from one compartment to two leads to increases in both entropy and internal energy, with their contributions to the free energy change acting in opposition. Conversely, in the reverse transition from the evenly distributed state to the single-compartment–full-CNT states, both entropy and internal energy decrease, but their contributions to the free energy change remain opposite. Similar examples of opposing thermodynamic changes have been widely observed in protein–ligand binding, molecular docking, and protein unfolding, as discussed in the context of EEC in the Introduction.

**Figure 8:**
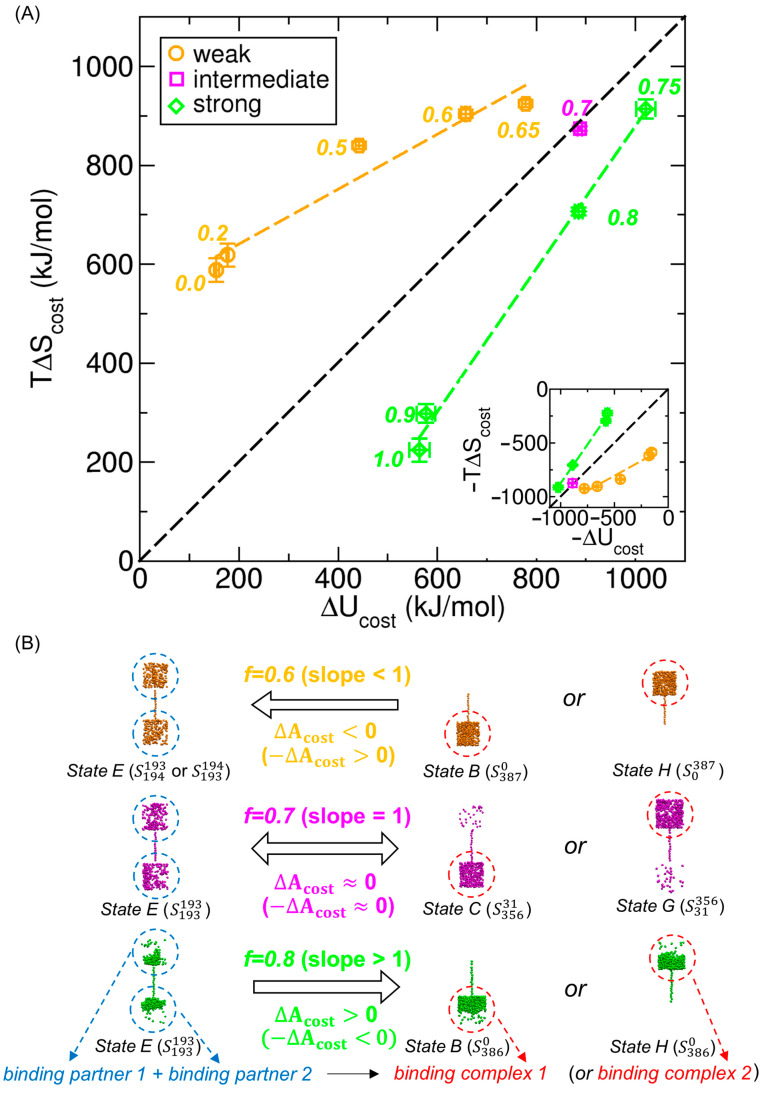
(**A**) Correlations between $∆U_{cost}$ and $T∆S_{cost}$ for different values of the interaction strength f, spanning weak (f< 0.7; orange), intermediate (f= 0.7; magenta), and strong (f> 0.7; green) regimes. All data points lie in the first quadrant, indicating that both contributions exhibit compensatory behavior. Colored dashed lines represent linear regressions for the weak (orange) and strong (green) interaction regimes. The slopes and correlation coefficients are 0.555 and 0.968 for the weak regime, and 1.444 and 0.998 for the strong regime, respectively. The black dashed line has a slope of 1 and represents perfect compensation. Each data point is labeled with its corresponding *f* value. Vertical and horizontal error bars indicate the standard deviations of $∆U_{cost}$ and $T∆S_{cost}$, respectively. The inset shows $-T∆S_{cost}$ versus $-∆U_{cost}$, as discussed in Section 3.6 in the context of molecular binding. (**B**) Thermodynamic favorability of transport to State E for representative interaction strengths. In the weak interaction (f= 0.6), forward transport (i.e., the transition from States B or H to State E) is thermodynamically favorable. In the intermediate interaction case (f= 0.7), forward and reverse transport occur reversibly. In the strong-interaction case (f= 0.8), reverse transport (from State E to States B or H) is favorable. The forward and reverse transport correspond to dissociation and association, respectively, in the context of molecular binding (see Section 3.6). For example, at  f= 0.8, two binding partners (blue circles) associate to form a complex (red circle). Molecules inside the CNT (carbon nanotube) are neglected, as they are few in number relative to the rest.

To more directly examine whether compensation exists and to quantify its extent, we plot $T∆S_{cost}$ versus $∆U_{cost}\;$ for various f values, as shown in Figure 8A. All data points lie in the first quadrant ($∆U_{cost}>\;$0 and $T∆S_{cost}>$ 0), confirming the presence of compensation. Moreover, in both the weak- and strong-interaction regimes, the data follow nearly linear trends with positive slopes, indicating strong correlation between the two contributions, as shown by the regression lines in Figure 8A.

However, the compensation in these regimes is not perfect, as the slopes differ from unity. Specifically, for weak interactions (f< 0.7), $∆U_{cost}$ increases more rapidly with f than $T∆S_{cost}$ does (see Figure 7A), i.e., $d\left(∆U_{cost}\right)/df>\;d\left(T∆S_{cost}\right)/df$, which yields a slope $d\left(T∆S_{cost}\right)/d\left(∆U_{cost}\right)<\;1$, as represented by the orange dashed line in Figure 8A. At the transition point (f= 0.7), $∆U_{cost}$ and $T∆S_{cost}\;$ become nearly equal, as indicated by the magenta square. For strong interactions (f> 0.7), both $∆U_{cost}$ and $T∆S_{cost}$ decrease as f increases (see Figure 7A), but $T∆S_{cost}$ decreases more rapidly than $\;∆U_{cost}$. This results in a slope $d\left(T∆S_{cost}\right)/d\left(∆U_{cost}\right)>\;1$, as illustrated by the green dashed line in Figure 8A.

The change in the compensation slope with f reflects the phase-dependent nature of the compensation effect and is associated with the change in $∆A_{cost}$. In the gas-like phase (weak interactions), entropy dominates at f = 0 (the nonpolar case), where $T∆S_{cost}>\;∆U_{cost}$ and thus $∆A_{cost}<$ 0. As f increases, both contributions grow, but $T∆S_{cost}$ grows more slowly than $∆U_{cost}$ (slope < 1), leading to a gradual shift of the free energy cost toward zero. Finally, at an intermediate interaction strength (f≈ 0.7), $T∆S_{cost}$ becomes nearly equal to $∆U_{cost}$, and the system reaches a gas–liquid transition crossover where $∆A_{cost}\approx$ 0. Beyond this point, in the liquid-like phase (strong interactions), both terms decrease, but $T∆S_{cost}$ decreases more rapidly than $∆U_{cost}$ (slope > 1), so internal energy becomes dominant, and $∆A_{cost}>\;$0.

Furthermore, in our system, the phase itself determines whether the slope in Figure 8A—relating $T∆S_{cost}$ to $∆U_{cost}$—is greater than, less than, or approximately equal to 1, and the slope, in turn, reflects the phase. Specifically, the slope is less than 1 in the gas-like phase, greater than 1 in the liquid-like phase, and close to 1 at the gas–liquid transition near f=0.7. If the slope were greater than 1 in the gas-like phase, the regression (orange) line would eventually intersect the diagonal (black dashed; slope = 1) line as f decreases, implying that weaker interactions promote a transition toward the liquid phase—an unphysical situation. Conversely, in the liquid-like phase, if the slope were less than 1, the regression (green) line would intersect the diagonal as *f* increases, suggesting that stronger interactions promote a transition toward the gas phase—another unphysical scenario.

Additionally, the diagonal line in Figure 8A marks the boundary between gas-like and liquid-like regimes. Points above it indicate gas-like behavior, where $∆A_{cost}$ is negative and the transition from the single-compartment–full-CNT states (States B or H) to the evenly distributed state (State E) is favorable. Points below correspond to liquid-like behavior, where $∆A_{cost}$ is positive and the transition is unfavorable. These trends are summarized in Figure 8B, including the case of $∆A_{cost}\approx \;$0 on the diagonal.

### 3.6. Extension of Energy–Entropy Compensation Analysis to Molecular Binding

Enthalpy (or energy)–entropy compensation is widely discussed in the context of molecular binding, particularly in studies of molecular recognition, docking, and drug design. From this perspective, the molecular transport process studied here can be analogously interpreted as a binding process. Specifically, the evenly distributed state (e.g., State E in Figure 2) and the single-compartment–full-CNT state (e.g., State B or H in Figure 2) correspond to the dissociated and associated states, respectively, in a molecular binding scenario.

In molecular binding, the transition of interest is from the dissociated (e.g., State E) to the associated state (e.g., States B or H), which is the reverse of the molecular transport direction. To clarify this analogy, the bottom panel in Figure 8B illustrates a binding reaction: binding partner 1 + binding partner 2 → binding complex. Here, two binding partners correspond to the two groups of molecules enclosed by blue dashed circles in the dissociated state (State E), and the binding complex corresponds to the single group of molecules enclosed by a red dashed circle in the associated state (States B or H). For this analogy, we neglect the molecules inside the CNT because they are few in number relative to the rest of the molecules. Accordingly, the relevant thermodynamic quantities for binding—namely, the free energy ($∆A_{dissociated\to associated}$), the energetic contribution ($∆U_{dissociated\to associated}$), and the entropic contribution ($T∆S_{dissociated\to associated}$)—are obtained by reversing the signs of the corresponding values computed for transport in Section 3.5: $∆A_{dissociated\to associated}=-∆A_{cost}$, $∆U_{dissociated\to associated}=-∆U_{cost}$, and $T∆S_{dissociated\to associated}=-T∆S_{cost}$. As a result, both $∆U_{dissociated\to associated}$ and $T∆S_{dissociated\to associated}$ are negative, as shown in the inset of Figure 8A.

A key distinction between our model and conventional binding models is that here, association occurs between two groups of many molecules interacting via nonbonded interactions, rather than between two distinct molecular entities—such as a ligand and a receptor—as is typically in molecular binding. Due to this many-body nature, our model exhibits phase behavior governed by the interaction strength f, which naturally influences the compensation characteristics, as discussed in Section 3.5.

As expected from the sign reversal, the trend in the inset of Figure 8A shows all data points now lying in the third quadrant. Again, a strong linear correlation is observed in both weak (f< 0.7) and strong (f> 0.7) interaction regimes. The conventional understanding of the compensation effect typically includes such linear enthalpy–entropy (or energy–entropy) relationships. In fact, some studies [57,61] define the compensation effect purely based on this linearity, whereas others [52,68] emphasize the signs of the changes to determine whether compensation occurs.

Following the compensation between the entropic and energetic components, the residual contribution—their difference—ultimately dictates the direction of the free energy change. As discussed in Section 3.5, the dominant contribution, and thus the residual, depends on f: the residual is entropic for f< 0.7 and energetic for f> 0.7, and nearly zero at f= 0.7. In this respect, weak-interaction cases (f< 0.7) are not relevant to molecular association, as dissociation is thermodynamically favored and no aggregation occurs (see top panel of Figure 8B). In contrast, strong-interaction cases (f> 0.7) support aggregation and are more representative of molecular binding behavior, as often observed in EEC analyses of molecular recognition and docking (see bottom panel of Figure 8B). In the intermediate case (f= 0.7), both association and dissociation are thermodynamically accessible (see middle panel of Figure 8B).

A straightforward graphical criterion for detecting energy–entropy compensation on a thermodynamic basis is whether data points and their regression line in the T∆S versus ∆U plot fall within the first quadrant (x> 0, y> 0) or the third quadrant (x< 0, y< 0). If so, both contributions share the same sign and compensation is present—as is the case in Figure 8A.

Additionally, if data points and their regression line lie on the diagonal (slope = 1), the system exhibits perfect compensation. This occurs only in the intermediate case (f= 0.7). In contrast, the weak- and strong-interaction regimes show regression slopes of 0.555 and 1.444, respectively, with corresponding correlation coefficients are 0.968 and 0.998. These values confirm strong, but non-perfect, linear compensation in both regimes.

In summary, the slope and position of the regression line in the T∆S versus ∆U plot provide clear insights into the nature of energy–entropy compensation. When phase behavior plays a role, these graphical criteria help determine whether the dominant contribution is energetic (slope > 1) or entropic (slope < 1), whether association (slope > 1) or dissociation (slope < 1) is favored, and whether the system behaves in a liquid-like (slope > 1) or gas-like (slope < 1) manner. This analysis illustrates how the energy–entropy compensation framework developed for molecular transport can be effectively extended to molecular binding phenomena.

## 4. Conclusions

In this study, we investigate the thermodynamics of molecular transport between two compartments connected by a nanochannel, focusing on how the interaction strength between molecules affects the entropic and energetic changes that together determine the free energy change. The free energy changes (PMF profiles) used for this analysis were obtained in our previous study [15]. By varying the interaction strength from small values (corresponding to gas-like behavior) to large values (characteristic of liquid-like behavior) and decomposing the free energy change into energetic and entropic contributions, we find that the free energy change due to molecular transport is energetically driven in liquid-like systems (strong interactions), whereas in gas-like systems (weak interactions), it is primarily driven by entropy.

To further understand the interplay between energy and entropy, we examine the correlation between the two contributions. We observe a linear relationship between contributions that share the same sign—indicative of an energy–entropy compensation effect—in both the strong- and weak-interaction regimes. In the strong-interaction regime, molecules tend to aggregate, lowering internal energy, while reducing translational and rotational entropy. In contrast, in the weak-interaction regime, the interactions are too weak to hold the molecules together; as a result, the molecules tend to disperse, gaining entropy through increased freedom of motion, while simultaneously increasing internal energy. These changes lead to opposing contributions to the free energy change, resulting in energy–entropy compensation. Therefore, our results provide clear evidence that, at least within our system, energy–entropy compensation is both present and systematic, supported by detailed thermodynamic analysis rather than correlation alone.

Interestingly, our transport model conceptually resembles molecular binding, where the single-compartment–full-CNT state corresponds to an associated state and the evenly distributed state represents a dissociated state. In this analogy, strong interactions in a liquid-like phase favor association, whereas weak interactions in a gas-like phase favor dissociation. Intermediate strengths lead to reversible association in a liquid–gas transition-like phase.

Quantitatively, the compensation effect is reflected in the approximately constant slope of the plot of entropic changes (T∆S) versus energetic changes (∆U). This plot describes the transition from a dissociated state to an associated state under various interaction strengths, with data points lying in either the first or third quadrants. While the exact origin of the slope in our system remains unclear, our analysis reveals that a slope greater than 1 corresponds to spontaneous association, whereas a slope less than 1 indicates that dissociation is thermodynamically favored. Only when the slope equals 1 do association and dissociation become thermodynamically reversible. Thus, in our model, the slope serves as a useful thermodynamic criterion for determining whether aggregation is favorable in molecular binding processes.

Although our present model is well suited for studying the thermodynamics of transporting all molecules from one compartment into an initially empty one—a setup relevant to nanotechnology and nanoscale engineering—it differs from more realistic systems such as biological environments, which involve inhomogeneous ion concentrations, diverse nanochannel architectures, variable solvent densities, and applied electric fields. It would be interesting, in future work, to incorporate each of these elements individually to investigate how they influence transport behavior, its underlying thermodynamics, and ultimately the EEC effect.

To build on these ideas and more directly address the extent to which the observations in this work can be generalized beyond our simplified model, we discuss the following points. Given the simplicity of our system, we are able to analyze the complete thermodynamic behavior of molecular binding derived from molecular transport in a closed and controlled setting under NVT conditions. However, realistic molecular binding systems are typically open (e.g., constant chemical potential (μ) and pressure (p) conditions), involve greater complexity (e.g., large and flexible molecules), and often feature partial observability of the components (e.g., two binding molecules in an effective field). Although our model is limited to binding transitions observed in molecular transport of simple molecules, the methodology developed here would be generally applicable to other realistic systems. For instance, the graphical method developed in our simplified model—based on the slope in T∆S versus ∆H plots—could potentially be applied to more complex systems, although the specific values may differ. Moreover, while the present study uses interaction strength as the perturbation to probe the compensation effect, other types of perturbations—such as changes in temperature, pressure, molecular size, functional group, or even combinations thereof—can also be applied. Using our model as a reference system and systematically varying such thermodynamic or perturbative conditions could help bridge the gap between idealized models and realistic systems. Furthermore, the observed slope in the compensation plot may vary depending on system-specific characteristics arising from underlying microscopic details. For example, in T∆S versus ∆H plots, the slope for *n*-alkane solvation across different chain lengths is approximately 0.546 [52,60], whereas the slope for protein unfolding across various proteins is approximately 0.963 [52,88]. This variation underscores the system-dependent nature of enthalpy (or energy)–entropy compensation and merits further investigation.

## Data Availability

The original contributions presented in this study are included in the article. Further inquiries can be directed to the corresponding author.
